# Correlation between molar activity, injection mass and uptake of the PARP targeting radiotracer [^18^F]olaparib in mouse models of glioma

**DOI:** 10.1186/s13550-022-00940-9

**Published:** 2022-10-09

**Authors:** Chung Ying Chan, Samantha L. Hopkins, Florian Guibbal, Anna Pacelli, Julia Baguña Torres, Michael Mosley, Doreen Lau, Patrick Isenegger, Zijun Chen, Thomas C. Wilson, Gemma Dias, Rebekka Hueting, Véronique Gouverneur, Bart Cornelissen

**Affiliations:** 1 CRUK/MRC Oxford Institute for Radiation Oncology, Department of Oncology, University of Oxford, Old Road Campus Research Building, Off Roosevelt Drive, Oxford, OX3 7LJ UK; 2 Department of Chemistry, Chemistry Research Laboratory, University of Oxford, 12 Mansfield Road, South Parks Road, Oxford, OX1 3TA UK; 3 Department of Nuclear Medicine and Molecular Imaging, University Medical Centre Groningen, University of Groningen, Groningen, The Netherlands

**Keywords:** PARP, Olaparib, [^18^F]olaparib, Glioblastoma, PET

## Abstract

**Purpose:**

Radiopharmaceuticals targeting poly(ADP-ribose) polymerase (PARP) have emerged as promising agents for cancer diagnosis and therapy. PARP enzymes are expressed in both cancerous and normal tissue. Hence, the injected mass, molar activity and potential pharmacological effects are important considerations for the use of radiolabelled PARP inhibitors for diagnostic and radionuclide therapeutic applications. Here, we performed a systematic evaluation by varying the molar activity of [^18^F]olaparib and the injected mass of [^Total^F]olaparib to investigate the effects on tumour and normal tissue uptake in two subcutaneous human glioblastoma xenograft models.

**Methods:**

[^18^F]Olaparib uptake was evaluated in the human glioblastoma models: in vitro on U251MG and U87MG cell lines, and in vivo on tumour xenograft-bearing mice, after administration of [^Total^F]olaparib (varying injected mass: 0.04–8.0 µg, and molar activity: 1–320 GBq/μmol).

**Results:**

Selective uptake of [^18^F]olaparib was demonstrated in both models. Tumour uptake was found to be dependent on the injected mass of [^Total^F]olaparib (µg) but not the molar activity. An injected mass of 1 μg resulted in the highest tumour uptake (up to 6.9 ± 1.3%ID/g), independent of the molar activity. In comparison, both the lower and higher injected masses of [^Total^F]olaparib resulted in lower relative tumour uptake (%ID/g; *P* < 0.05). Ex vivo analysis of U87MG xenograft sections showed that the heterogeneity in [^18^F]olaparib intratumoural uptake correlated with PARP1 expression. Substantial upregulation of PARP1-3 expression was observed after administration of [^Total^F]olaparib (> 0.5 µg).

**Conclusion:**

Our findings show that the injected mass of [^Total^F]olaparib has significant effects on tumour uptake. Moderate injected masses of PARP inhibitor-derived radiopharmaceuticals may lead to improved relative tumour uptake and tumour-to-background ratio for cancer diagnosis and radionuclide therapy.

**Supplementary Information:**

The online version contains supplementary material available at 10.1186/s13550-022-00940-9.

## Introduction

The important roles of poly(ADP-ribose) polymerase (PARP) in regulation of multiple cellular processes, including response to DNA damage [1] together with PARP expression profiles in various types of cancers, make PARP-targeting a promising strategy for cancer diagnosis and therapy [2]. To date, five PARP inhibitors (olaparib, rucaparib, niraparib, talazoparib and veliparib) have been approved by the United States Food and Drug Administration (FDA) as single agents for the treatment of cancers with defects in homologous recombination (HRD), such as those bearing *BRCA1/2* mutations. Additionally, a variety of PARP-targeting radiopharmaceuticals have been developed, based on the chemical structures of PARP inhibitors, to visualise PARP expression in tumours or act as radionuclide therapy agents for cancer treatment [2–5]. The biggest advantage of radiolabelled PARP inhibitors is that they allow non-invasive visualisation and quantification of PARP expression in patients, which may aid patient stratification for treatment with PARP inhibitors, drug-target engagement and therapeutic monitoring. Furthermore, among PARP-targeting radiopharmaceuticals, [^18^F]olaparib [6], [^18^F]talazoparib [3, 4] and [^18^F]rucaparib [5, 7] are the ^18^F-labelled isotopologues of their parent compounds with identical chemical structures, which are expected to display the same pharmacokinetic and pharmacodynamic profiles in vivo, hence providing useful clinical information for PARP inhibition therapy in cancers. Several radiolabelled PARP inhibitors, [^18^F]FTT (rucaparib-based) and [^18^F]PARPi (olaparib-based), have demonstrated their clinical utility for imaging different types of cancers [8–13].

These radiopharmaceuticals are derived from bioactive or pharmaceutical molecules, it is important to administer an appropriate amount of mass and radioactivity to allow for accurate imaging of the subject without causing any unwanted pharmacological effects [14]. Therefore, injected mass and molar activity of the radiopharmaceuticals are a crucial factor. Despite the development of several PARP-targeting imaging agents, pharmacological effects, such as PARP trapping, allosteric effects and other potential nonlinear effects of these compounds have not yet been investigated. Radiolabelled PARP-targeting agents may induce different allosteric or reverse allosteric effects, which will have an impact on stabilising and trapping PARP protein and the PARP inhibitor on DNA. This effect of PARP trapping will prolong retention of the radiolabelled compound, and besides is known to induce DNA double-strand breaks (DSB) by stalling and collapsing replication forks or accelerating fork elongation [2]. The formation of DSBs may in turn lead to upregulation of PARP expression for DNA damage response, thereby affecting the uptake and accumulation of the PARP-targeting agents in the tissue-of-interest. Therefore, the injected formulation (injected mass and molar activity) of PARP-targeting radiopharmaceuticals may have a direct influence on their distribution and accumulation in tumour and organs, especially spleen and liver, which have higher expression levels of PARP1, 2 and 3 (The European Bioinformatics Institute: www.ebi.ac.uk). By far, only one study from Salinas et al*.* showed the effects of the molar activity on the uptake of a PARP inhibitor-derived compound, [^131^I]-I2-PARPi, in tumour and other organs, whereby lower molar activity of [^131^I]-I2-PARPi (0.185 GBq/μmol) resulted in higher uptake in tumour and other organs, compared to the higher molar activity of [^131^I]-I2-PARPi (9.25 GBq/μmol) [15]. The mechanism of which remains to be elucidated.

Glioblastoma (GBM) is one of the tough-to-treat cancers. Several GBM models have been used in different evaluation studies of PARP targeting radiopharmaceuticals, and their PARP expression profiles have also been determined [15–17]. In addition, recent studies have shown the promising potential of PARP inhibitors in chemo-/radiotherapy to improve treatment efficacy in GBM models [18, 19]. Here, we evaluated the effects of injected mass (μg) and molar activity (GBq/μmol) on the biodistribution and accumulation of one such PARP-targeting radiopharmaceutical, [^18^F]olaparib, in two GMB models: U251MG and U87MG, and investigated the expression profile of PARP isoforms following exposure to the PARP inhibitor. [^18^F]olaparib was demonstrated to have good selectivity towards PARP1, 2 and 3 in pancreatic ductal adenocarcinoma (PDAC) preclinical models [6], making [^18^F]olaparib a good candidate as an imaging tool to study the effects of PARP inhibitor on PARP isoform expression. The aim of this study is to perform a systematic evaluation on the effects of varying injected mass and molar activity on tumour uptake of [^18^F]olaparib and PARP expression. This has important implications on the clinical translation of PARP-targeting radiopharmaceuticals for diagnostic imaging and radionuclide therapy.

## Methods

Unless otherwise noted, all reagents were purchased from Sigma-Aldrich and used without further purification.

### Cell culture

U251MG and U87MG human malignant glioma cells were kindly donated by Prof. Nicola Sibson at University of Oxford and authenticated by STR profiling. Cells were maintained in high glucose Dulbecco’s Modified Eagle Medium (DMEM) supplemented with 10% foetal bovine serum (FBS, Gibco), 2 mM L-glutamine, 100 units/mL penicillin and 0.1 mg/mL streptomycin (Gibco). Cells were grown under a humidified environment at 37 °C and 5% CO_2_. Cells were harvested or passaged 1:10 to T175 flask using trypsin–EDTA solution. Cells were used no more than 20 passages following resuscitation from liquid nitrogen storage, and tested regularly for the absence of mycoplasma.

### PARP isoform expression in human malignant glioma cells

Relative expression of PARP isoforms in U251MG and U87MG cell lines was determined by western blots of cell lysates. Total protein lysates were prepared at 4 °C using RIPA buffer (50 mM Tris—pH 8.0, 1% NP40, 0.5% sodium deoxycholate, 0.1% sodium dodecyl sulphate, 150 mM sodium chloride, cOmplete™ protease inhibitor cocktail [Sigma-Aldrich]). Western blot was performed using the following antibodies (Atlas Antibodies-Sigma Aldrich, UK): anti-human PARP rabbit polyclonal antibodies at 1:500 dilution: anti-PARP1 antibody (HPA045168), anti-PARP2 antibody (HPA052003) and anti-PARP3 antibody (HPA067657) secondary goat anti-rabbit-HRP antibody (1:3000 dilution) (R&D Systems HAF008). Full details of procedures and protocols are provided in the Additional file 1.

### In vitro uptake and binding selectivity of [^18^F]olaparib

[^18^F]Olaparib was synthesised as previously described [6, 20]. [^Total^F]Olaparib indicates [^18^F + ^19^F]olaparib. In some cases, radiolabelled olaparib was mixed with unlabelled olaparib to lower the molar activity. Uptake and selectivity of [^18^F]olaparib was determined in U251MG and U87MG cells. Aliquots of cells were seeded (5 × 10^4^ cells/well, in 100 μL growth medium) in 96-well plates and allowed to adhere for at least 4 h. Cells were washed and exposed to unlabelled olaparib and talazoparib (100 μM, in a total of 100 μL growth medium) for 30 min at 37 °C. Additionally, [^18^F]olaparib ([^Total^F]olaparib final concentration: 0.1 μM, molar activity = 11.8 GBq/μmol) was added, and the cells were incubated at 37 °C for a further hour. This was followed by removal of the cell culture medium, and washing of cells with PBS. Cells were lysed using RIPA buffer for 15 min at room temperature, and the amount of ^18^F in the cell lysates and supernatant was measured using an automated gamma counter (PerkinElmer).

### Quantification of PARP isoform expression in U87MG cells treated with olaparib

Relative expression of PARP isoforms was determined using flow cytometry in U87MG cells after exposure to unlabelled olaparib. U87MG cells (1 × 10^6^ cells/well) were seeded in 96-well plates and exposed to olaparib (final concentration: 0–1 μM in 200 μL growth medium) for 3 h at 37 °C. Cells were washed with FACS buffer (PBS, 2% FBS, 1 mM EDTA, 0.1% NaN_3_) and centrifugation at 500 × g for 5 min. Immunostaining was performed using the Foxp3/transcription factor staining buffer set (eBioscience™, USA). Intracellular staining was conducted in permeabilisation buffer for 30 min at 4 °C in the dark separately using the following antibodies: AF488-conjugated anti-PARP-1 (1:100; sc-80070), AF594-conjugated anti-PARP-2 (1:100; sc-393310) and AF488-conjugated anti-PARP-3 (1:100; sc-390771) from Santa Cruz Biotechnology Inc. Fixable viability dye ef780 (1:4000; eBioscience™; 65-0865-14) was used for live and dead cells discrimination. Fixation of immunostained cells was performed for 15 min at room temperature. Flow cytometry was conducted on the CytoFLEX benchtop flow cytometer (Beckman Coulter, USA), with appropriate laser and filters, positive and negative controls. Data were analysed using FlowJo™ (Tree Star Inc., BD Biosciences, USA).

### PET/CT imaging and biodistribution of [^18^F]olaparib in Balb/c xenograft mice

All animal procedures were performed in accordance with the United Kingdom Home Office’s Guidance on the Operation of Animals (Scientific Procedures) Act 1986 and the Animal Research: Reporting of In Vivo Experiments (ARRIVE) guidelines. Local ethical committee approval was obtained (PPL PA1B5C52F, University of Oxford). Female athymic Balb/c nu/nu (CAnN.Cg-*Foxn1*^*nu*^/Crl) mice, aged 5–7 weeks, were purchased from Charles Rivers (UK). Animals were housed in IVC cages, up to 6 mice per cage, in an artificial day–night cycle facility. Food and water were provided ad libitum.

For tumour implantation, cells were harvested using trypsin–EDTA, washed twice with PBS, and reconstituted in PBS:Matrigel® Matrix High Concentration (1:1). Cell suspensions (U251MG: 5 × 10^6^ cells/ 100 μL and U87MG: 1 × 10^7^ cells/100 μL) were injected subcutaneously in the front left flank of mice.

Animals were administered with different masses of [^Total^F]olaparib: 0.04, 0.5, 1.0, 4.0 or 8.0 μg in 100 μL of PBS, molar activity = 1–320 GBq/μmol) by intravenous (i.v.) injection via the lateral tail vein. To evaluate the selectivity of tumour uptake, an excess of unlabelled olaparib (20 μg) was co-administered as a blocking agent. PET/CT images were acquired 120 min later using a MILabs VECTor^4^ camera, equipped with an ultra-high resolution rat/mouse 1.8 mm collimator, followed by a cone-beam CT scan (55 kV, 0.19 mA) for anatomical reference and attenuation correction. Animal was anaesthesied by 4% isoflurane gas (0.5 L/min O_2_) and maintained at 2.5% at 37 °C throughout the duration of image acquisition. PET images were reconstructed using U-SPECT-Rec3.22 software (MILabs, Utrecht, The Netherlands), using a pixel-based algorithm of 6 subsets, 4 iterations and 0.8 mm voxel size for fluorine-18 (energy window settings 477.9–584.1 keV). The reconstructed PET and CT images were viewed and analysed using PMOD v.3.37 (PMOD Technologies, Zurich, Switzerland).

Following PET/CT acquisition or 120 min after radiolabelled compound administration, animals were culled experimentally by Schedule 1 cervical dislocation. Organs, tissues and blood were harvested for gamma counting based on the percentage of the injected dose per gram of tissue (%ID/g), using a HIDEX automated gamma counter.

Ex vivo localisation of [^18^F]olaparib in U251MG and U87MG xenografts was determined using autoradiography performed on frozen tumour sections (10 μm). Uptake in U87MG tumours was further compared to immunohistochemistry staining for PARP1, 2 and 3 and compared to sections harvested from tumour xenografts, which was exposed to external beam radiation (10 Gy, 2 Gy/min, Gulmay 320 kV irradiator. The radiation set-up allowed irradiation of the right hind quarter, including the tumour and right leg, only.). Full details of the procedures and protocols are provided in the Additional file 1.

### Statistical analyses

All data were obtained at least in duplicate. Statistical analysis and nonlinear regression were performed using GraphPad Prism v8 (GraphPad Software, San Diego, CA, USA). Data were tested for normality and analysed as appropriate by unpaired t-test with Welch’s correction or ANOVA with Dunnet’s post hoc test for multiple comparisons. The results are reported as mean ± one standard deviation, unless otherwise stated.

## Results

### Uptake of [^18^F]olaparib in GBM cells is highly selective

**Fig. 1:**
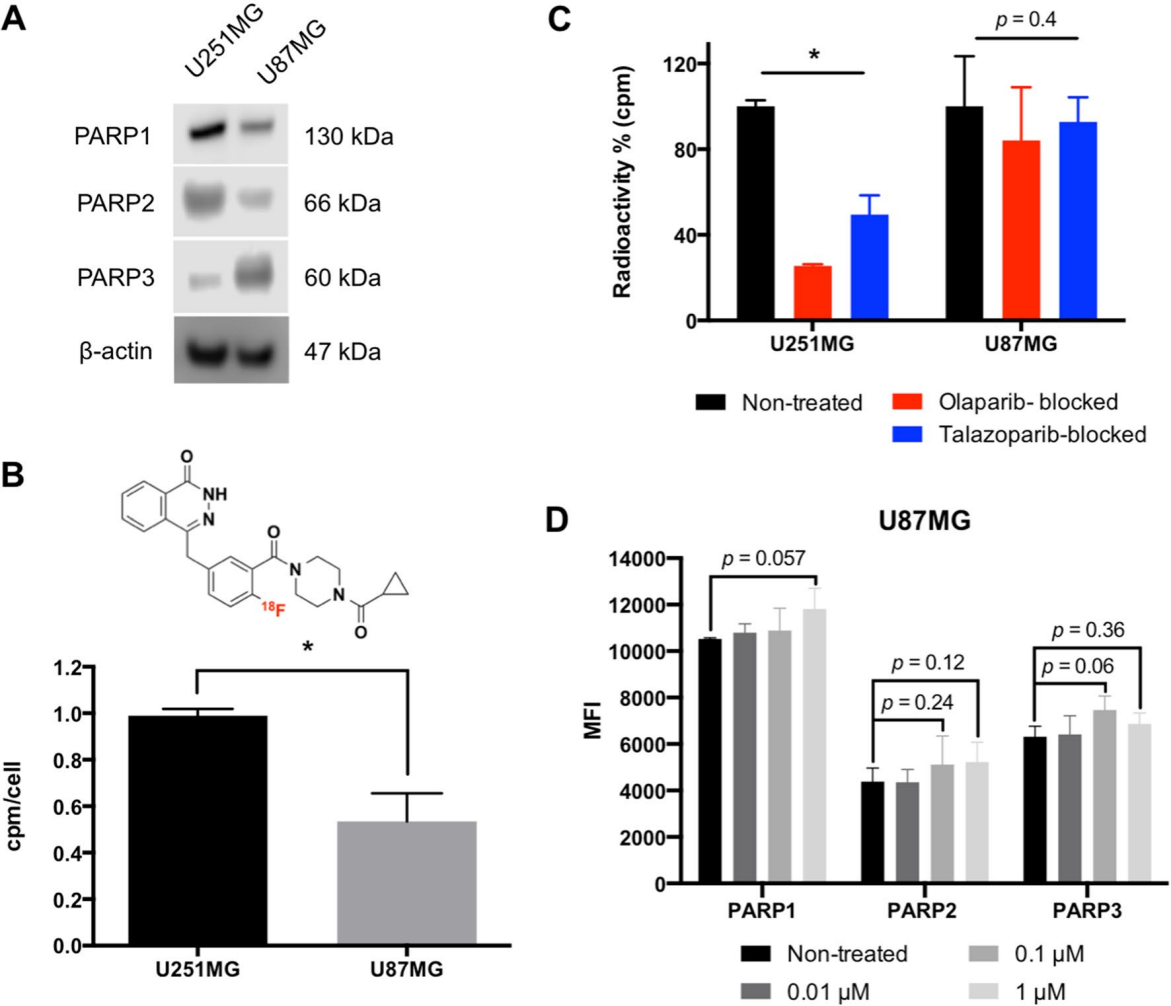
**A** Western blotting for PARP1-3 expression in U251MG and U87MG lysates. **B** Chemical structure of [^18^F]olaparib, and its uptake in U251MG and U87MG cells ([^Total^F]olaparib final concentration: 0.1 μM, molar activity = 11.8 GBq/μmol). **C** Blocking of [^18^F]olaparib uptake in U251MG and U87MG cells by unlabelled olaparib or talazoparib. **D** Mean fluorescence intensity (MFI) for PARP1-3 of U87MG cells treated with unlabelled olaparib (0–1 μM), assessed by flow cytometric analysis. *n* = 3 independent experiments. Representative histogram frequency is presented in Additional file 1: Fig. S1. Asterisks indicate levels of significance: *P < 0.05

Immunoblotting showed PARP1 and PARP2 levels to be higher in U251MG cells compared to U87MG, while the opposite was observed for PARP3 (Fig. 1A). This corresponded to the higher uptake of [^18^F]olaparib in U251MG cells compared to U87MG cells (Fig. 1B). The addition of an excess amount of unlabelled olaparib and talazoparib was able to significantly reduce the uptake of [^18^F]olaparib in U251MG cells cell line (*P* < 0.05), but not in U87MG cells (*P* = 0.4) (Fig. 1C). The degree of binding selectivity of [^18^F]olaparib observed in two different cells is intriguing; however, we hypothesise that this may be due to differences in intrinsic expression of PARP in U251MG and U87MG cell lines, off-target binding of PARP inhibitors, PARP trapping effects and induction of PARP expression caused by exposure to PARP inhibitor, thereby inducing the formation of DSBs and resulting in upregulation of PARP expression for DNA damage response. We subsequently investigated if treatment with increasing amounts of olaparib could induce up-regulation of PARP expressions in U87MG cells. Flow cytometric analysis revealed a trend that PARP1, 2 and 3 expression levels were slightly upregulated upon exposure to up to 1 μM of olaparib although not statistically significant. Further exploration on the mechanisms of protein-PARP inhibitor interaction is warranted.

### In vivo uptake of [^18^F]olaparib in U251MG and U87MG tumour xenografts reveals a dependency on injected mass and higher PARP expression

**Fig. 2:**
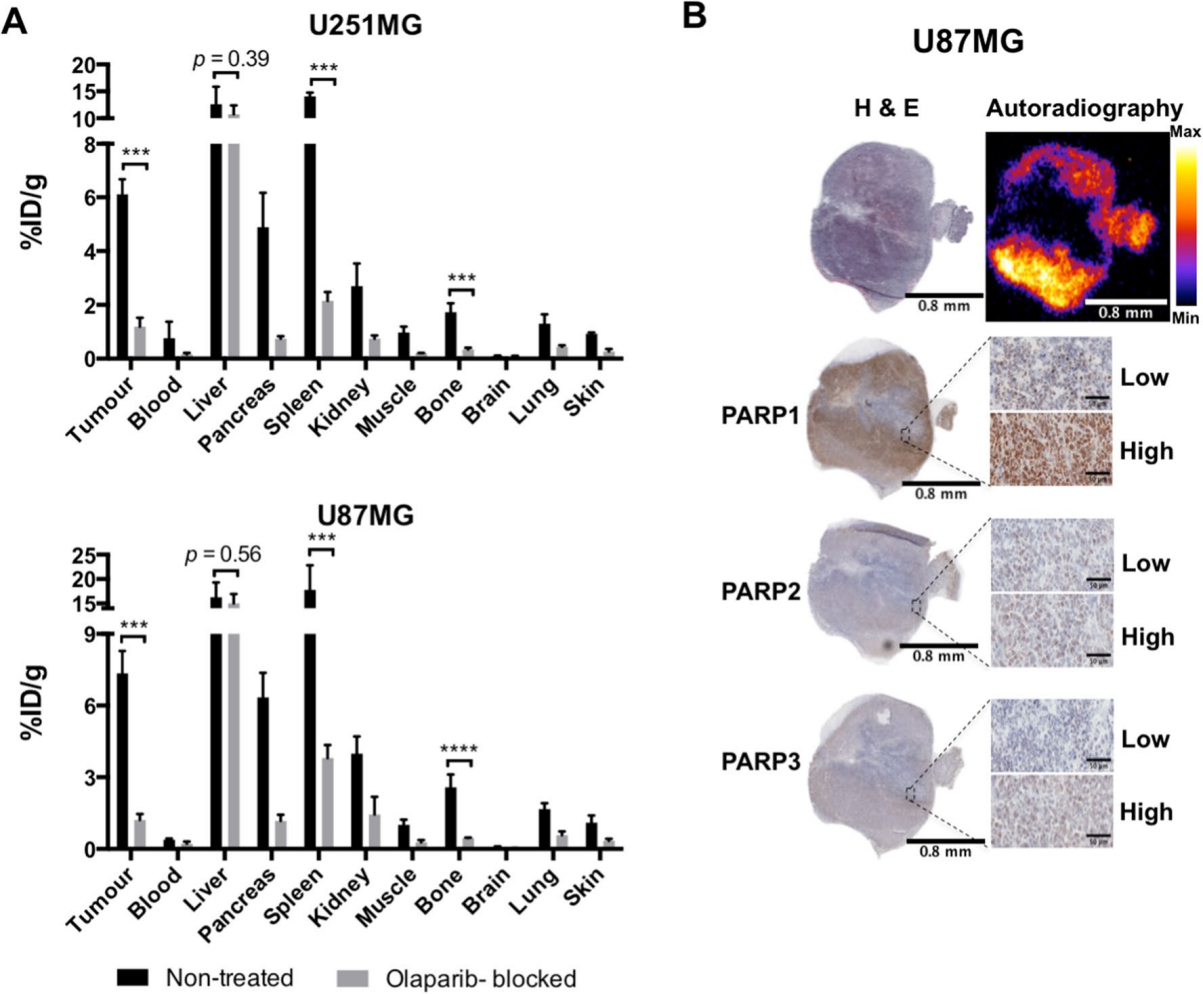
**A** Biodistribution in selected tissues in U251MG or U87MG xenograft-bearing mice, 120 min after i.v. injection of [^18^F]olaparib (0.28–0.31 MBq, molar activity = 1.9 GBq/μmol) (*n* = 3/group), with or without an excess of unlabelled olaparib (20 μg). Further data are presented in Additional file 1: Table S1 and Figure S2. **B** Autoradiography of U87MG tumour sections showing ^18^F localisation, and immunohistochemical staining of adjacent U87MG tumour sections showing PARP1, 2 and 3 expression. Asterisks indicate levels of significance: ***P < 0.001; ****P < 0.0001

Following i.v. administration of [^18^F]olaparib (0.31 MBq, molar activity = 1.9 GBq/μmol), biodistribution was similar to that described before, whereby high uptake of the radiopharmaceutical was detected in the liver, gallbladder, large/small intestine and caecum due to the hepatobiliary clearance of [^18^F]olaparib (Additional file 1: Table S1) [6]. Tumour uptake of [^18^F]olaparib in U87MG xenografts was higher than that in U251MG (7.4 ± 0.9%ID/g vs. 6.1 ± 0.5%ID/g, respectively, *P* < 0.001) at 2 h after i.v. administration (Fig. 2A). [^18^F]Olaparib tumour uptake could be blocked by co-administration of an excess of unlabelled olaparib in either tumour model (from 6.1 ± 0.5%ID/g and 7.4 ± 0.9%ID/g to 1.2 ± 0.3%ID/g and 1.2 ± 0.2%ID/g, in U251MG and U87MG xenografts, respectively; *P* < 0.001), and their PARP expressing organs, such as spleen, pancreases and liver, indicating selective uptake. Less blocking effect observed in liver, comparing other organs, which may be due to the [^18^F]olaparib undergoing hepatobiliary clearance. Ex vivo analysis combining immunohistochemical staining and autoradiography of U87MG tumour tissues further demonstrated that the uptake of [^18^F]olaparib correlated with PARP expression, predominantly of which are contributed by PARP1 expression (Fig. 2B). Therefore, the heterogeneity of PARP expression found in U87MG xenograft tumour may have resulted in the uneven uptake of [^18^F]olaparib, suggesting that tumour heterogeneity has a significant impact on the uptake of radiolabelled PARP inhibitors, including those used for radionuclide therapy.

**Fig. 3:**
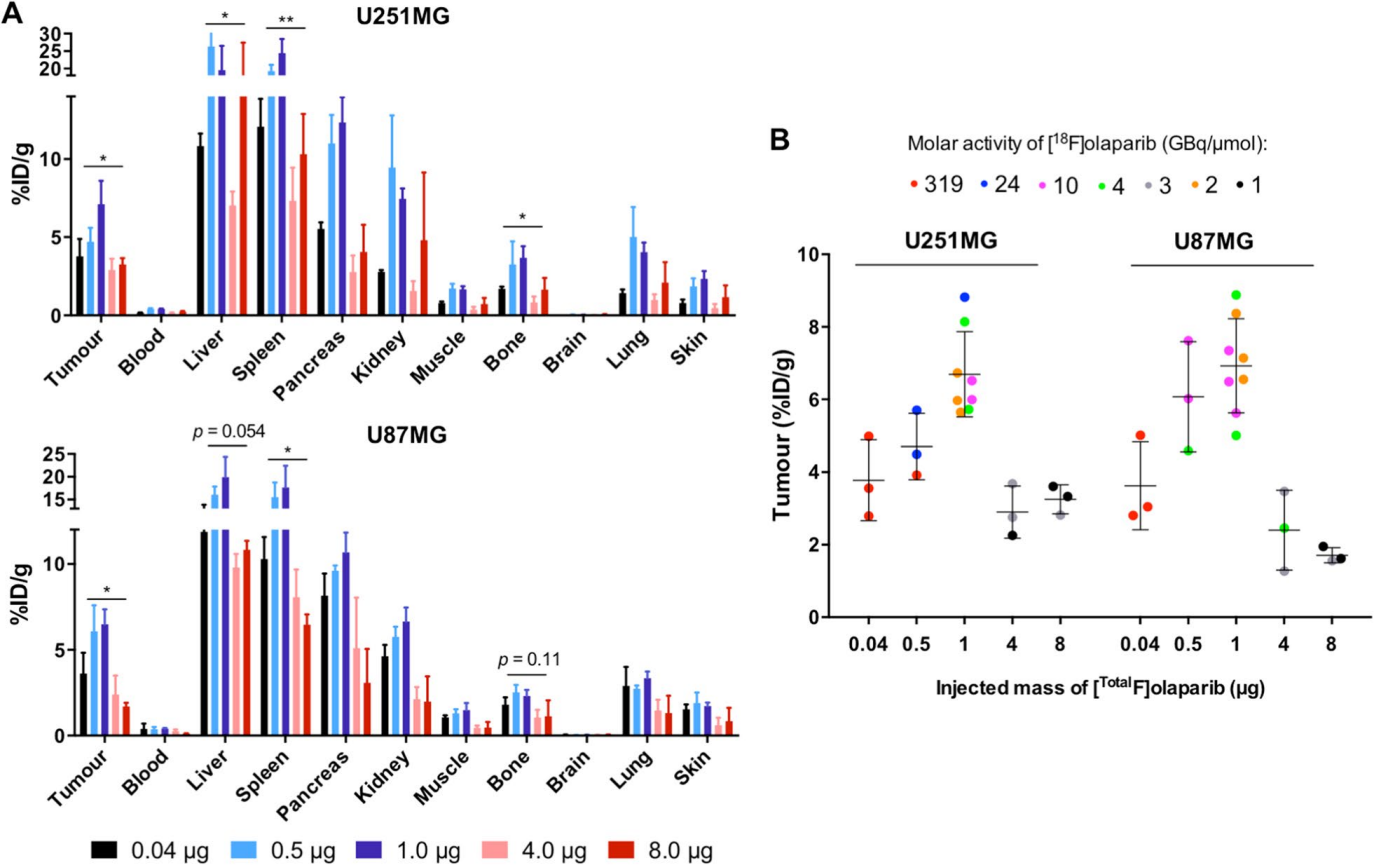
**A** Biodistribution of [^18^F]olaparib in selected tissues in U251MG (top) or U87MG (bottom) xenografts bearing-mice, 120 min after i.v. administration of [^Total^F]olaparib (0.04–8.0 μg, [^18^F]olaparib: 0.28–13.89 MBq) with various molar activities (1–320 GBq/μmol), (*n* = 3/group). Full biodistribution data are presented in Additional file 1: Tables S2–S3. **B** Tumour uptake of [^18^F]olaparib at various injected masses (μg) and molar activities (also see Additional file 1: Table S4). Asterisks indicate levels of significance: *P < 0.05; **P < 0.01

To investigate the effect of varying the injected mass of [^Total^F]olaparib on [^18^F]olaparib tumour uptake, we administered 0.04, 0.5, 1.0, 4.0 or 8.0 μg of [^Total^F]olaparib (injected activity: 0.28–13.89 MBq, molar activity = 1–320 GBq/μmol). Tumour accumulation of [^18^F]olaparib was significantly affected by the injected mass of the radiotracer, but not by the molar activity (GBq/μmol) (Fig. 3) or total injected radioactivity (MBq) (Additional file 1: Fig. S3) or tumour size (g) (Additional file 1: Fig. S4). The lowest injected mass (0.04 μg) in this study resulted in tumour uptake of 3.8 ± 1.1 and 3.6 ± 1.2%ID/g for U251MG and U87MG xenografts, respectively, similar to previous reports on other radiolabelled PARP inhibitors [16, 17]. However, increasing injected masses resulted in a marked increase (0.5 and 1.0 μg), followed by a decrease (4.0 and 8.0 μg) in tumour uptake (measured as %ID/g) for both tumour xenografts (Fig. 3B). This trend of [^18^F]olaparib uptake was also observed in other high PARP expressing organs such as spleen, liver and bone for either xenograft models (Fig. 3A), suggesting that injected mass of the radiolabelled PARP inhibitors is an important consideration for dosing, formulation, therapeutic index and organ toxicity in PARP-targeting radionuclide therapy.

### PARP expression increases after olaparib administration

**Fig. 4:**
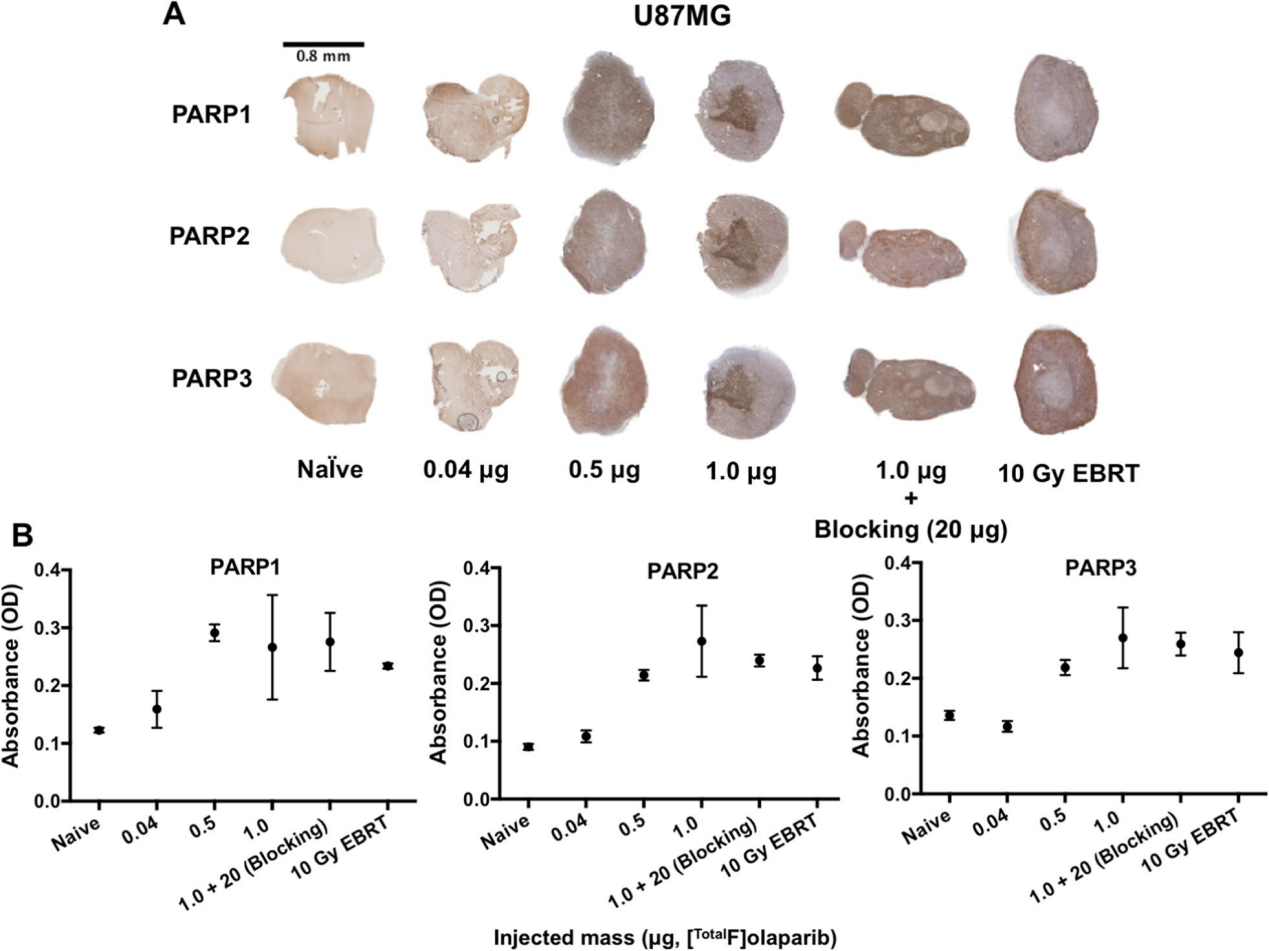
**A** Immunohistochemistry staining for PARP1, 2, or 3 in U87MG xenograft tumour sections harvested from animals 120 min after i.v. administration of varying masses (μg) of [^Total^F]olaparib or after irradiation (10 Gy). **B** Semi-quantification of PARP staining. Each data point represents 3 different analysed fields randomly selected on the tumour section (also see Additional file 1: Tables S5, S6)

To further investigate the correlation between injected mass, [^18^F]olaparib tumour uptake and target expression, we employed immunohistochemistry to semi-quantitatively compare PARP1, 2, and 3 expression in U87MG xenografts (Fig. 4). Low injected mass (0.04 μg) of [^Total^F]olaparib had no significant impact on PARP expression in the U87MG xenografts, while increasing the injected mass of [^Total^F]olaparib from 0.5 to 21.0 μg led to a marked increase in PARP1-3 expression in the U87MG xenografts, in agreement with the observations of U87MG cells treated with unlabelled olaparib (Fig. 1D). External beam irradiation (10 Gy) similarly increased PARP1-3 expression. These observations suggested that low injected mass of radiolabelled PARP inhibitors may not alter target expression, and treatment of either olaparib (> 0.04 μg) or EBRT may induce DNA damages, hence resulting in the upregulation of PARP expression and uptake of radiolabelled PARP inhibitor in the tumour.

## Discussion

PARP inhibitors have seen a steep increase in attention over the past years. Several inhibitors are now approved for use as monotherapy for HR-deficient cancers, with multiple others at various stages of development. Concurrently, a multitude of PARP inhibitor-based radiolabelled compounds have been reported for use in molecular imaging and radionuclide therapy [2]. The relatively high tumour uptake of radiolabelled PARP inhibitors, together with clear differentiation between PARP over-expressing tumour tissue and surrounding normal tissues creates new avenues to measure PARP expression levels, tumour detection, patient selection, dose optimisation and radionuclide therapy targeting PARP. Because of the ability of PARP inhibitors to trap PARP-DNA complexes, or allosterically alter PARP binding to DNA, or vice versa, nonlinear effects may exist. Therefore, the injection formulation (including injected mass and molar activity) of radiolabelled PARP inhibitors is crucial to ensure accurate diagnosis or improved therapeutic index of radionuclide treatments. Additionally, the injection formulation may affect the uptake of radiolabelled PARP inhibitor in tumours and normal organs. Here, we used [^18^F]olaparib, a PARP-targeted PET imaging agent which previously demonstrated successful imaging of PARP in human PDAC xenograft models [6], to investigate the effects of injected mass (μg) and molar activity of [^18^F]olaparib on tumour uptake and target expression in glioblastoma mouse models. This is to enable the optimisation of imaging contrast and radionuclide radiation absorbed dose.

In vitro uptake of [^18^F]olaparib was shown to be higher in U251MG compared to U87MG cells, which aligns with PARP1 and 2 expression levels obtained in these cell lines from western blotting. The uptake in these cells was selective, as suggested by blocking studies using an excess of unlabelled olaparib and talazoparib. These findings confirmed our earlier study in pancreatic cancer cells [6] and proved PARP1 and 2 isoforms but not PARP3 as the major contributors for [^18^F]olaparib uptake in these cells. Conversely, the in vivo uptake of [^18^F]olaparib in U251MG (6.1 ± 0.5%ID/g) and U87MG (7.4 ± 0.9%ID/g) xenograft tumours did not align with the expression levels of PARP1-3 from western blot analysis. Similar uptake of [^18^F]olaparib in both glioblastoma xenograft models suggested that the tumour uptake of [^18^F]olaparib is governed by more than PARP expression levels alone, and caution should be taken when interpreting quantification of imaging data. Higher uptake in tumour tissue does not necessitate higher PARP expression levels. It has, however, been described that in tumours that respond to PARP inhibitor treatment, the level of uptake (in vitro) is correlated to cell viability, as a proxy for tumour growth inhibition [21]. Our results suggested that this may not necessarily be the case in vivo, and detailed studies of this use of radiolabelled PARP imaging agents are warranted.

Consistent with our previous results and observations reported for other PARP inhibitor-derived compounds, [^18^F]olaparib displayed a hepatobiliary clearance pattern, with high uptake in spleen, pancreas and bone, which was influenced significantly by varying the administered mass. The latter may be explained by the PARP1-3 expression in those three organs, where the higher amounts of unlabelled olaparib was able to block uptake of [^18^F]olaparib. Differences in uptake in normal tissues, on the other hand, may be due to an altered pharmacokinetic profile because of the higher total dose of olaparib, affecting clearance from the blood (Fig. 3A). Taken together, the relatively high tumour uptake of [^18^F]olaparib and the tumour-to-brain ratio (Additional file 1: Fig. S6) in U251MG and U87MG xenografts illustrate the potential of [^18^F]olaparib in glioma (Additional file 1: Figure S7) or neuroblastoma imaging, given existing evidence that olaparib can be delivered through the disrupted blood–brain barrier associated with these lesions [22].

Autoradiography, followed by immunohistochemical staining showed uneven distributions of both [^18^F]olaparib and PARP1-3 in U87MG tumour xenograft tissue (Figs. 2B and 4A), indicating the effects of tumour heterogeneity on target expression and [^18^F]olaparib uptake. Tumour heterogeneity, thus, should be taken into consideration when quantifying target expression and engagement in tumours for PARP imaging, and dosimetry estimations for PARP inhibitor-based radionuclide therapy. This demonstrates a clear need to investigate the relationship between tumour heterogeneity, PARP expression and radiolabelled PARP inhibitors to aid in the accurate diagnosis of PARP expression on PET imaging and optimisation of radionuclide therapy based on PARP radiopharmaceuticals.

As the effective dose and radiation absorbed doses in normal tissues are relevant for radionuclide therapy to achieve optimal outcome [23], we evaluated the effects of injected mass (μg) and molar activity on tumour and organ uptake of [^18^F]olaparib, as a model for other radiolabelled PARP inhibitors. Our in vivo data showed that varying the injected mass of [^Total^F]olaparib from 0.04 to 8.0 μg had significant effects on both tumour and normal tissue uptake. This effect was independent of molar activity (1–320 GBq/μmol) or injected radioactivity (0.28–13.89 MBq. An intermediate injected mass (1 μg), regardless of molar activity, showed the highest relative uptake of [^18^F]olaparib with good tumour-to-blood ratio in both U87MG and U251MG xenografts, indicating the fast clearance of this radiotracer and considering as a suitable does for in vivo imaging purpose, whereas lower injected masses (0.04 and 0.5 μg) resulted in relative tumour uptake that was similar to that reported for other radiolabelled olaparib-based imaging agents [16]. Interestingly, larger injected masses (4.0 and 8.0 μg) also resulted in lower uptake of [^18^F]olaparib in the tumour plausibly caused by self-blocking, although the average PARP1 levels in all tissues are usually relatively high. Together with flow cytometry (Fig. 1D) and immunohistochemistry studies (Fig. 4) on the U87MG models, our results suggested that exposure to olaparib affects the expression levels of PARP1-3 in vitro and in vivo, which may further influence the uptake levels. Caution should therefore be taken in using radiolabelled PARP inhibitors at higher injected masses to quantify target engagement. On the other hand, optimisation of injected mass may be advisable when radiolabelled PARP inhibitors are employed for radionuclide therapy.

We hypothesise that this Goldilocks effect (not too little, not too much) may be explained by a combination of three possible mechanisms: (1) Induction of PARP expression in the tumour of animals that were administered with higher masses of olaparib. Our results suggest that the amount of olaparib, despite being far lower than that used for therapy, could markedly influence PARP1-3 expression levels, which could in turn influence radiolabelled PARP inhibitor uptake levels in the tumour, despite the short timeframe (2 h post-injection); (2) Saturation of PARP inhibitor binding pockets occurs at higher injected masses. Comparison of Fig. 3C and the amount of [^Total^F]olaparib accumulated in tumour (in pmol per gram of tumour tissue, Additional file 1: Fig. S5) indicates that the amount of olaparib per gram in tumour tissue does not follow the same trend as the radioactivity (MBq) per gram of tumour. Higher injected masses of [^Total^F]olaparib (4 and 8 μg) gave lower relative tumour uptake, similar to the tumour uptake from blocking study (21 μg, Fig. 2A), and therefore supports saturation of olaparib binding sites with higher injected masses.; and/or (3) Alteration of the metabolisation and/or pharmacokinetics of [^18^F]olaparib with higher injected masses. We show that differences in tumour-to-organ ratios (Additional file 1: Fig. S6) are significant in elimination organs across administered amounts in each xenograft model, indicating that the injected mass may affect the pharmacokinetics of [^Total^F]olaparib, hence influencing tumour uptake.

Although blocking of the binding pocket and effects on pharmacokinetics are expected, the induction of PARP expression, at least within this time frame, was less so. We propose that increased PARP expression levels may be a result of triggered DNA damage response, which can be attributed to olaparib binding and trapping of PARP to DNA and other unknown factors. Several studies have reported that PARP1-DNA trapping induces conversion of SSBs into DSBs after replication, thereby enhancing DSB levels, although these studies were performed over time scales of several days [24, 25]. The higher DSB levels would result in increased levels of DNA damage response signalling, thus contributing to an increase in expression levels of PARP1, 2 and 3. However, further investigation to gain better insights on the correlation between PARP inhibitor dose and resulting PARP expression level is necessary.

Finally, our results suggested that the injected mass of radiolabelled PARP inhibitors can be optimised, since the credo ‘higher molar activity and lower injected mass is better’ does not seem to hold for targeting PARP, at least in the models we have evaluated here. This may have important implications for the different uses of radiolabelled PARP inhibitors, whether for imaging or radionuclide therapy. An appropriate formulation of radiolabelled PARP inhibitor may improve image contrast and improve the delivery of radiopharmaceuticals to tumours for PARP-targeting radionuclide therapy.

## Conclusion

Tumour uptake of radiolabelled PARP inhibitors, such as [^18^F]olaparib, is governed by more than PARP expression levels alone. We observed that [^18^F]olaparib uptake in malignant glioma xenografts was affected by injected mass, which we attribute to a combination of induction of PARP target expression, saturation of PARPi binding pockets and alteration of olaparib clearance. Taken together, tumour uptake of radiolabelled PARP inhibitors may be enhanced by optimising the injected mass, which could improve PARP imaging accuracy for diagnostic purposes and make radionuclide therapy more efficacious.

## Supplementary Information

**Additional file 1**. Supplementary information.
