# Supplementary material for: Correlation between molar activity, injection mass and uptake of the PARP targeting radiotracer [18F]olaparib in mouse models of glioma
Source: EJNMMI Res. 2022 Oct 9;12:67. doi: 10.1186/s13550-022-00940-9 (PMC9548459; doi:10.1186/s13550-022-00940-9)
Supplement: Supplementary file 1 — Additional file 1. Supplementary information. [file 13550_2022_940_MOESM1_ESM.docx]

**Supplemental information**

**Correlation between molar activity, injection mass and uptake of the PARP targeting radiotracer [^18^F]olaparib in mouse models of glioma**

Chung Ying Chan^1^*, Samantha Hopkins^1^*, Florian Guibbal^1,2^, Anna Pacelli^1^, Julia Baguña Torres^1^, Michael Mosley^1^, Doreen Lau^1^, Patrick Isenegger^2^, Zijun Chen^2^, Thomas Wilson^2^, Gemma Dias^1^, Rebekka Hueting^1^, Véronique Gouverneur^2,†^, and Bart Cornelissen^1,3,†^

*^1^* Oxford Institute for Radiation Oncology, Department of Oncology, University of Oxford, Oxford, OX3 7DQ, UK.

*^2^* Department of Chemistry, Chemistry Research Laboratory, University of Oxford, 12 Mansfield Road, Oxford, OX1 3TA, UK.

*^3^* Department of Nuclear Medicine and Molecular Imaging, University Medical Centre Groningen, University of Groningen, Groningen, The Netherlands.

* These authors contributed equally on this work.

**Supplemental Methods:**

Unless otherwise noted, all reagents were purchased from Sigma-Aldrich and used without further purification.

**Western blot of glioblastoma cell lysates**

Western blots of cell lysates were performed to determine relative expression of PARP isoforms. Total protein preparations were produced at 4°C using RIPA buffer (50 mM Tris – pH 8.0, 1% NP40, 0.5% sodium deoxycholate, 0.1% sodium dodecyl sulphate, 150 mM sodium chloride, cOmplete^TM^ protease inhibitor cocktail [Sigma-Aldrich]). Cell lysates were isolated by centrifugation after lysis through a 21G hypodermic syringe. Bicinchoninic acid (BCA) colorimetric assay (Thermo Fisher) was used to normalise for total protein content. Thirty micrograms of cell lysate samples were run on a 4-12% NuPAGE Bis-Tris SDS gel (Novex) in MES running buffer (Novex), before semi-dry protein transfer (iBlot2) to a PVDF membrane. Membranes were washed with PBS-Tween20 (0.1%) for 10 min and blocked using 5% skim milk in PBS-Tween20 (0.1%) for 120 min. Anti-human PARP rabbit polyclonal antibodies at 1:500 dilution: anti-PARP1 antibody (HPA045168), anti-PARP2 antibody (HPA052003), and anti-PARP3 antibody (HPA067657) were incubated with membrane in 2.5% skim milk in PBS-Tween20 (0.1%) at 4°C overnight. The membranes were then washed for 3 x 10 min in PBS-Tween20 (0.1%) and incubated with the secondary goat anti-rabbit-HRP antibody (1:3000 dilution) (R&D Systems HAF008) at RT for 1 h, washed three time for 10 min with PBS-Tween20 (0.1%), and incubated with SuperSignal^TM^ West Pico PLUS Chemiluminescent substrate (Thermo-Fisher) for 5 min. Images were acquired using a Licor C-Digit.

**Autoradiography and Immunohistochemical staining**

U251MG and U87MG xenograft tissues were flash-frozen using isopentane on dry ice and sectioned (10 µm) using a Leica CM1950 cryostat. Tissue sections were thaw-mounted onto Superfrost PLUS glass microscope slides (Menzel-Glaser, Thermo Scientific) and allowed to dry at room temperature (RT). The slides were then fixed in 10% NBF for 10 min at RT, dried, and exposed to a storage phosphor screen (PerkinElmer, Super Resolution, 12.5 x 25.2 cm) in a standard X-ray cassette for 15 h at 4°C. The phosphor screen was then imaged using a Cyclone® Plus Storage Phosphor System (PerkinElmer).

For tissues that were not used in autoradiography, after imaging and automated gamma counting, selected tissues from mice were fixed in 10% neutral buffered formalin overnight. Tissue was then flash frozen and stored at -80°C overnight. Frozen tissue was sectioned (10 µm) using a Leica CM1950 cryostat. Tissue sections were thaw-mounted onto Superfrost PLUS glass microscope slides (Menzel-Glaser, Thermo Scientific) and allowed to dry at RT. The slides were then stored at 4°C until used.

**Immunohistochemical staining**

For immunohistochemical staining, slides were placed at RT for 5-10 mins to thaw. Slide incubation procedures were performed in glass Coplin type jars. The sections were dehydrated and rehydrated by sequential immersion in ethanol at 100% (twice), 70% and 50%, for 3 min at each concentration, with a final 1 min wash in water. The slides were then immersed in citrate buffer (10 mM sodium citrate pH 6.0) with 0.05% tween-20, and antigen retrieval was performed in an antigen-decloaking chamber (Biocare Medical) at 125°C for 2.5 min, then 95°C for 1 min. The slides were allowed to cool, then placed on ice.

The sections were processed using the EnVision FLEX DAB+ Substrate Chromogen System (Dako Omnis; Agilent Stockport UK). Tissue sections were blocked in Peroxidase and Alkaline Phosphatase Blocking Reagent (S2003) for 15 min at RT, then incubated overnight at 4°C in tris-buffered saline buffer (TBS) with 1% bovine serum albumen (BSA) containing the following Atlas Antibodies (Sigma Aldrich, UK): anti-human PARP rabbit polyclonal antibodies at 1:150 dilution: anti-PARP1 antibody (HPA045168), anti-PARP2 antibody (HPA052003), and anti-PARP3 antibody (HPA067657). Following this incubation, the tissues were washed five times (5 min each) in phosphate-buffered saline (PBS), incubated for 30 min with Envision rabbit/mouse HRP polymer (K4065), and developed for 2.5 min using 1:50 dilution of DAB+ 2-component substrate (K3467). The sections were then washed in water twice (5 min each), and the nuclei counter-stained for 1 min in aqueous haematoxylin. This was followed by a 3 min wash in water, and four separate incubations in 100% ethanol (30 sec each), and two incubations in xylene (30 sec each). Coverslips were then mounted on DPX mounting media, and the slides left to set, and stored at 4°C.

Positive PARP staining in the tissue sections appeared brown, and the nuclei were counter-stained pink by the haematoxylin. Images were acquired using a brightfield microscopy at 40x magnification (MLeica biosystem ScanScope CS2, Milton Keynes UK). PARP expression was enumerated manually on each tumor section using at least 3 randomly selected fields of view section.

**Supplemental table:**

**Supplemental Table S1**: *Ex vivo* biodistribution of [^18^F]olaparib (0.28-0.31 MBq, molar activity = 1.9 GBq/μmol) in U87MG and U251MG xenograft bearing-mice (n=3/group) with or without the co-injection of unlabelled olaparib (20 µg) as blocking agent.

| Tumour Xenograft %ID/g | U87MG-blocked | | | U87MG-non block | | | U251MG-blocked | | | U251MG-non block | | |
| --- | --- | --- | --- | --- | --- | --- | --- | --- | --- | --- | --- | --- |
| Blood | 0.33 | 0.16 | 0.16 | 0.44 | 0.34 | 0.37 | 0.22 | 0.15 | 0.10 | 0.47 | 0.36 | 1.47 |
| Tumour | 1.14 | 1.03 | 1.49 | 7.14 | 6.55 | 8.37 | 1.54 | 0.89 | 1.14 | 5.97 | 6.73 | 5.64 |
| Heart | 0.44 | 0.26 | 0.31 | 1.01 | 0.70 | 1.02 | 0.21 | 0.23 | 0.40 | 0.83 | 0.68 | 0.63 |
| Lung | 0.75 | 0.44 | 0.55 | 1.88 | 1.39 | 1.73 | 0.39 | 0.45 | 0.50 | 1.44 | 1.56 | 0.91 |
| Liver | 16.35 | 15.93 | 12.82 | 19.56 | 13.67 | 15.61 | 12.39 | 9.13 | 10.74 | 14.92 | 14.05 | 8.95 |
| Spleen | 4.42 | 3.40 | 3.60 | 18.29 | 12.51 | 22.55 | 2.51 | 1.87 | 2.08 | 14.65 | 13.32 | 14.29 |
| Stomach | 2.28 | 1.24 | 3.51 | 3.99 | 2.68 | 5.83 | 0.80 | 2.10 | 2.23 | 4.23 | 3.43 | 3.28 |
| Large intestine | 19.59 | 19.71 | 15.62 | 38.43 | 27.12 | 26.34 | 31.34 | 90.56 | 88.47 | 45.69 | 58.84 | 45.24 |
| Small intestine | 72.15 | 57.42 | 52.18 | 29.03 | 24.09 | 29.89 | 40.38 | 15.62 | 18.95 | 24.14 | 16.82 | 17.60 |
| Pancreas | 1.39 | 0.90 | 1.25 | 7.25 | 5.24 | 6.55 | 0.83 | 0.76 | 0.65 | 5.56 | 5.70 | 3.42 |
| Kidney | 2.29 | 0.90 | 1.13 | 4.71 | 3.26 | 4.00 | 0.84 | 0.62 | 0.78 | 3.41 | 2.91 | 1.78 |
| Muscle | 0.37 | 0.28 | 0.19 | 1.19 | 0.77 | 1.07 | 0.20 | 0.20 | 0.22 | 0.80 | 0.91 | 1.22 |
| Skin | 0.41 | 0.26 | 0.37 | 1.40 | 0.80 | 1.12 | 0.29 | 0.35 | 0.17 | 0.97 | 0.93 | 0.87 |
| Fat | 0.22 | 0.24 | 0.33 | 5.59 | 1.56 | 0.68 | 0.23 | 0.19 | 0.31 | 0.80 | 1.00 | -- |
| Bone | 0.46 | 0.48 | 0.44 | 2.62 | 2.03 | 3.10 | 0.35 | 0.28 | 0.40 | 2.11 | 1.59 | 1.48 |
| Caecum | 22.09 | 27.96 | 99.56 | 61.44 | 61.39 | *--* | 81.85 | 184.08 | 175.74 | 95.13 | 96.66 | 131.24 |
| Gallbladder | 78.87 | 180.66 | 147.48 | *--* | 60.93 | 75.94 | 123.51 | 73.06 | 36.54 | 73.52 | 99.15 | 45.74 |
| Brain | 0.05 | 0.03 | 0.04 | 0.08 | 0.04 | 0.11 | 0.03 | 0.04 | 0.11 | 0.12 | 0.09 | 0.07 |

**Supplemental Table S2**: *Ex vivo* biodistribution of [^18^F]olaparib (%ID/g) in the U215MG xenograft bearing-mice (n=3/group), 120 min after i.v. administration of [^Total^F]olaparib (0.04-8.0 μg, [^18^F]olaparib: 0.28-13.89 MBq) with various molar activities (1-320 GBq/μmol).

| U215MG  Xenograft %ID/g | Injected mass ([^Total^F]olaparib) | | | | | | | | | | | | | | |
| --- | --- | --- | --- | --- | --- | --- | --- | --- | --- | --- | --- | --- | --- | --- | --- |
|  | 0.04 µg | | | 0.5 µg | | | 1.0 µg | | | 4.0 µg | | | 8.0 µg | | |
| Blood | 0.18 | 0.15 | 0.18 | 0.47 | 0.35 | 0.38 | 0.42 | 0.41 | 0.44 | 0.20 | 0.12 | 0.09 | *--* | 0.27 | 0.21 |
| Tumour | 3.56 | 2.79 | 4.98 | 5.70 | 4.49 | 3.92 | 8.82 | 6.52 | 5.99 | 3.68 | 2.76 | 2.26 | 2.82 | 3.33 | 3.61 |
| Heart | 0.78 | 0.68 | 0.80 | 3.36 | 2.49 | 1.68 | 2.55 | 2.30 | 1.95 | 0.57 | 0.42 | 0.26 | 1.91 | 0.79 | 0.57 |
| Lung | 1.68 | 1.21 | 1.37 | 6.97 | 4.95 | 3.12 | 4.72 | 3.86 | 3.56 | 1.39 | 0.86 | 0.72 | 3.59 | 1.49 | 1.20 |
| Liver | 11.16 | 9.92 | 11.41 | 35.27 | 24.47 | 19.22 | 27.16 | 13.45 | 18.05 | 7.96 | 6.99 | 6.18 | 28.73 | 13.84 | 10.18 |
| Spleen | 10.06 | 12.75 | 13.41 | 21.12 | 17.38 | 19.23 | 19.79 | 26.68 | 26.89 | 9.74 | 6.50 | 5.75 | 13.27 | 8.63 | 9.05 |
| Stomach | 1.09 | 0.76 | 1.56 | 4.70 | 2.23 | 1.93 | 2.90 | 25.75 | 2.20 | 3.71 | 0.57 | 1.22 | 8.11 | 2.13 | 0.50 |
| Large intestine | 13.32 | 49.46 | 57.79 | 8.78 | 10.88 | 27.82 | 10.44 | 4.27 | 20.61 | 18.72 | 8.59 | 42.58 | 11.87 | 9.01 | 38.73 |
| Small intestine | 19.33 | 11.75 | 14.22 | 39.85 | 41.78 | 23.56 | 35.99 | 36.80 | 33.69 | 21.55 | 12.18 | 12.23 | 21.00 | 61.98 | 44.69 |
| Pancreas | 5.38 | 5.23 | 6.01 | 10.48 | 13.03 | 9.50 | 14.16 | 11.09 | 11.78 | 3.93 | 2.50 | 1.88 | 6.06 | 2.97 | 3.14 |
| Kidney | 2.92 | 2.73 | 2.70 | 12.67 | 9.70 | 6.04 | 8.17 | 7.36 | 6.88 | 2.26 | 1.37 | 1.08 | 9.80 | 2.55 | 2.06 |
| Muscle | 0.82 | 0.70 | 0.87 | 1.99 | 1.77 | 1.42 | 1.89 | 1.54 | 1.59 | 0.57 | 0.29 | 0.26 | 1.17 | 0.60 | 0.42 |
| Skin | 0.97 | 0.56 | 0.86 | 2.00 | 2.28 | 1.27 | 2.42 | 1.82 | 2.79 | 0.77 | 0.32 | 0.33 | 1.99 | 0.98 | 0.55 |
| Fat | 0.31 | 1.63 | 0.74 | 0.87 | 3.09 | 1.96 | 1.05 | 1.49 | 2.09 | 0.95 | 0.30 | 0.24 | 7.82 | 0.44 | 0.63 |
| Bone | 1.55 | 1.68 | 1.85 | 4.22 | 3.99 | 1.58 | 3.10 | 3.45 | 4.51 | 1.22 | 0.49 | 0.79 | 2.38 | 1.68 | 0.88 |
| Caecum | 17.76 | 49.66 | 57.73 | 8.06 | 17.97 | 49.40 | 8.90 | 41.10 | 32.75 | 15.47 | 12.35 | 45.39 | 6.45 | 14.38 | 97.51 |
| Gallbladder | 25.47 | 24.40 | 16.25 | 58.89 | 55.32 | 82.25 | 41.83 | 84.21 | 86.78 | 12.89 | 17.72 | 20.49 | *--* | 51.95 | 40.69 |
| Brain | 0.02 | 0.02 | 0.02 | 0.05 | 0.04 | 0.06 | 0.05 | 0.04 | 0.04 | 0.03 | 0.02 | 0.01 | 0.09 | 0.03 | 0.02 |

**Supplemental Table S3**: *Ex vivo* biodistribution of [^18^F]olaparib (%ID/g) in the U87MG xenograft bearing-mice (n=3/group), 120 min after i.v. administration of [^Total^F]olaparib (0.04-8.0 μg, [^18^F]olaparib: 0.28-13.89 MBq) with various molar activities (1-320 GBq/μmol).

| U87MG  Xenograft %ID/g | Injected mass ([^Total^F]olaparib) | | | | | | | | | | | | | | |
| --- | --- | --- | --- | --- | --- | --- | --- | --- | --- | --- | --- | --- | --- | --- | --- |
|  | 0.04 µg | | | 0.5 µg | | | 1.0 µg | | | 4.0 µg | | | 8.0 µg | | |
| Blood | 0.76 | 0.23 | 0.21 | 0.47 | *--* | 0.29 | 0.41 | 0.45 | 0.38 | 0.38 | 0.23 | 0.21 | *--* | 0.11 | 0.13 |
| Tumour | 5.01 | 3.05 | 2.81 | 7.62 | 6.02 | 4.59 | 7.35 | 5.62 | 6.49 | 3.47 | 1.27 | 2.46 | 1.56 | 1.62 | 1.95 |
| Heart | 1.88 | 1.15 | 1.02 | 1.18 | 1.57 | 1.51 | 1.97 | 1.81 | 1.87 | 0.85 | 0.40 | 1.08 | 1.44 | 0.33 | 0.36 |
| Lung | 2.71 | 4.09 | 1.92 | 2.86 | 2.53 | 2.85 | 3.79 | 3.22 | 3.03 | 1.74 | 0.77 | 1.92 | 2.49 | 0.73 | 0.76 |
| Liver | 9.73 | 13.67 | 12.24 | 17.95 | 15.89 | 14.36 | 24.74 | 18.98 | 16.03 | 9.52 | 10.69 | 9.15 | 10.61 | 11.42 | 10.48 |
| Spleen | 8.86 | 11.35 | 10.65 | 18.15 | 16.48 | 11.98 | 19.13 | 12.42 | 21.50 | 7.32 | 6.97 | 9.90 | 6.74 | 6.88 | 5.77 |
| Stomach | 1.19 | 1.45 | 1.63 | 1.01 | 1.38 | 3.75 | 2.13 | 1.31 | 3.77 | 2.02 | 3.31 | 0.70 | 2.32 | 2.93 | 0.43 |
| Large intestine | 15.39 | 13.60 | 18.81 | 16.86 | 8.32 | 10.76 | 16.42 | 13.52 | 10.91 | 14.48 | *--* | 8.93 | 26.81 | *--* | *--* |
| Small intestine | 17.70 | 18.95 | 17.65 | 42.79 | 25.29 | 33.04 | 40.72 | 33.72 | 38.64 | 31.36 | 9.74 | 23.81 | 28.57 | 17.76 | 9.37 |
| Pancreas | 9.61 | 7.63 | 7.21 | 9.78 | 9.78 | 9.22 | 11.63 | 11.01 | 9.38 | 4.71 | 2.39 | 8.22 | 5.36 | 1.81 | 2.09 |
| Kidney | 5.39 | 4.26 | 4.24 | 5.17 | 5.76 | 6.35 | 7.58 | 6.23 | 6.15 | 2.78 | 1.37 | 2.23 | 3.68 | 1.18 | 1.12 |
| Muscle | 1.19 | 1.07 | 0.95 | 1.49 | 1.06 | 1.39 | 1.37 | 1.96 | 1.15 | 0.61 | 0.36 | 0.41 | 0.86 | 0.28 | 0.30 |
| Skin | 1.87 | 1.40 | 1.36 | 2.54 | 1.33 | 1.87 | 1.94 | 1.72 | 1.55 | 1.12 | 0.41 | 0.33 | 1.74 | 0.38 | 0.44 |
| Fat | 10.68 | 1.78 | 1.38 | 1.90 | 1.00 | 1.60 | 1.64 | 2.72 | 1.32 | 0.87 | 0.24 | 0.27 | 3.39 | 0.14 | 0.21 |
| Bone | 2.22 | 1.85 | 1.37 | 3.03 | 2.22 | 2.35 | 2.03 | 2.19 | 2.72 | 0.90 | 0.71 | 1.57 | 2.20 | 0.61 | 0.60 |
| Caecum | 13.32 | 19.64 | 40.32 | 33.53 | 14.75 | 11.90 | 8.08 | 19.42 | 18.01 | 15.07 | *--* | 15.66 | 10.28 | *--* | 91.52 |
| Gallbladder | 24.23 | 18.60 | 31.81 | 78.39 | 31.04 | 42.10 | -- | 35.81 | *--* | 22.84 | 25.78 | *--* | 10.93 | 28.20 | 32.95 |
| Brain | 0.07 | 0.03 | 0.03 | 0.03 | 0.04 | 0.04 | 0.05 | 0.05 | 0.03 | 0.05 | 0.02 | 0.02 | 0.07 | 0.01 | 0.02 |

**Supplemental Table S4**: Tumour uptake of [^18^F]olaparib at various injected masses. Error bars are mean ± SD.

| Tumour Xenograft %ID/g | Injected mass of [^Total^F]olaparib | | | | | |
| --- | --- | --- | --- | --- | --- | --- |
|  | 0.04 μg | 0.5 μg | 1.0 μg | 4.0 μg | 8.0 μg | 21.0 μg |
| U87MG | 3.6 ± 1.2 | 6.1 ± 1.5 | 6.9 ± 1.3 | 2.4 ± 1.1 | 1.7 ± 0.2 | 1.2 ± 0.2 |
| U251MG | 3.8 ± 1.1 | 4.7 ± 0.9 | 6.7 ± 1.2 | 2.9 ± 0.7 | 3.3 ± 0.4 | 1.2 ± 0.3 |

**Supplemental Table S5**: Summary of PARP1-3 expression in different organs. [www.ebi.ac.uk](http://www.ebi.ac.uk)

|  | Bone | Spleen | Pancreas | Lung | Skin | Intestine | Liver | Caecum |
| --- | --- | --- | --- | --- | --- | --- | --- | --- |
| PARP1 | high | high | mid | mid | mid | mid | mid | mid |
| PARP2 | mid | high | mid | high | mid | mid | mid | mid |
| PARP3 | low | low | low | low | mid | low | mid | low |

**Supplemental Table S6**: Comparisons of PARP mRNA expression levels in U251MG and U87MG cells. [www.ebi.ac.uk](http://www.ebi.ac.uk)

| Transcript Per Million | U251MG | U87MG |
| --- | --- | --- |
| PARP1 | 200 | 144 |
| PARP2 | 27 | 29 |
| PARP3 | 24 | 51 |

**Supplemental data:**

**
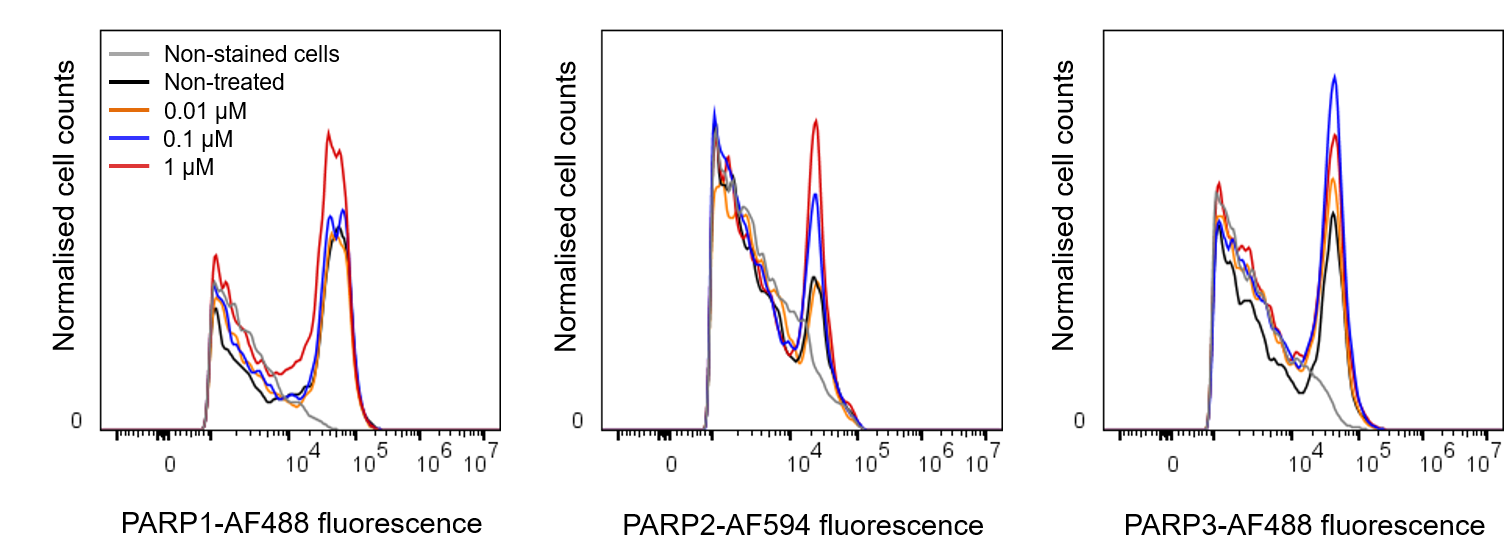
**

**Supplemental Fig. S1**: Representative histogram frequency probing for PARP1-3 expressions in U87MG cells after exposed to unlabelled olaparib (0-1 μM) for 3 h. n = 3 independent experiments.


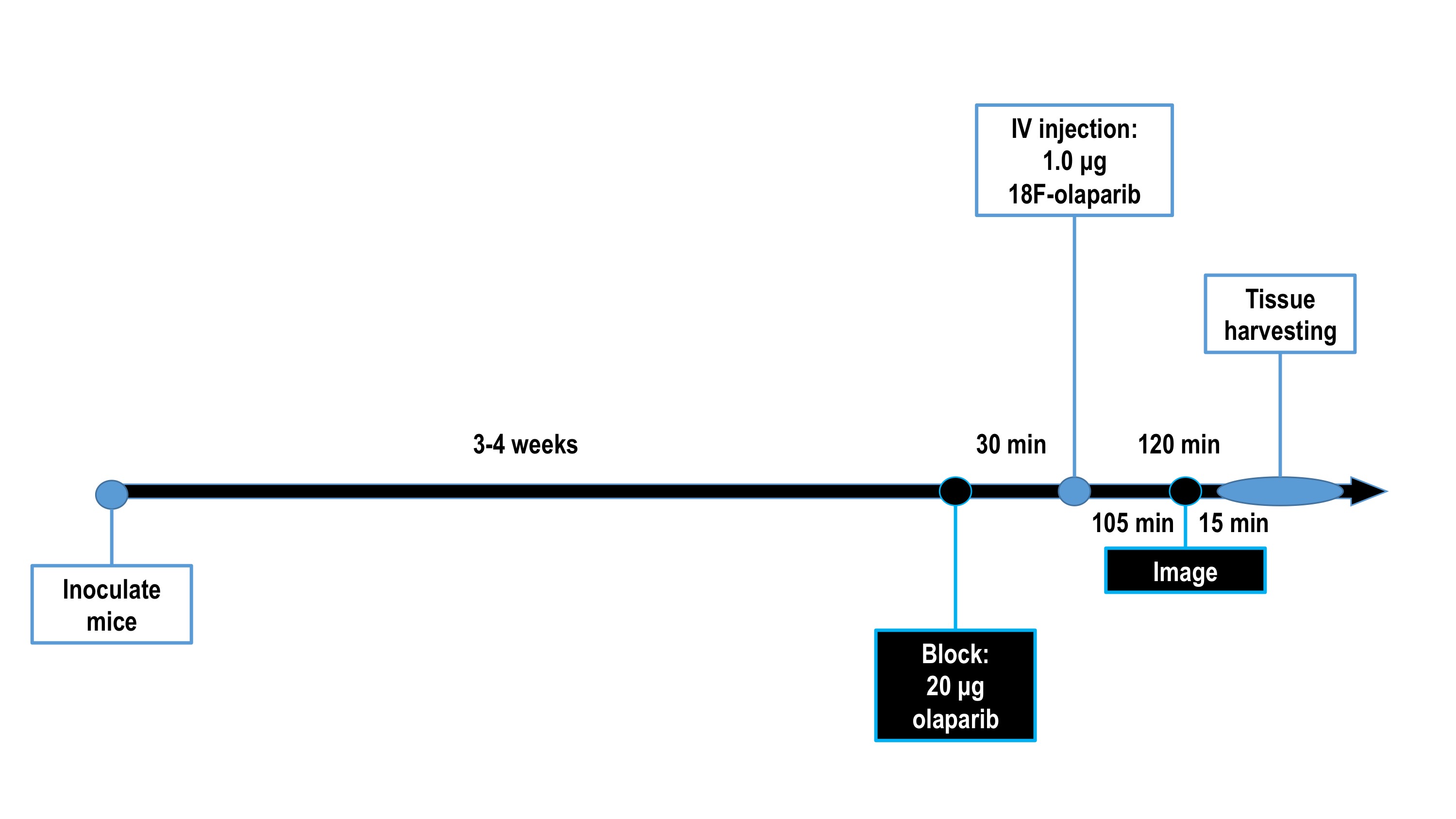


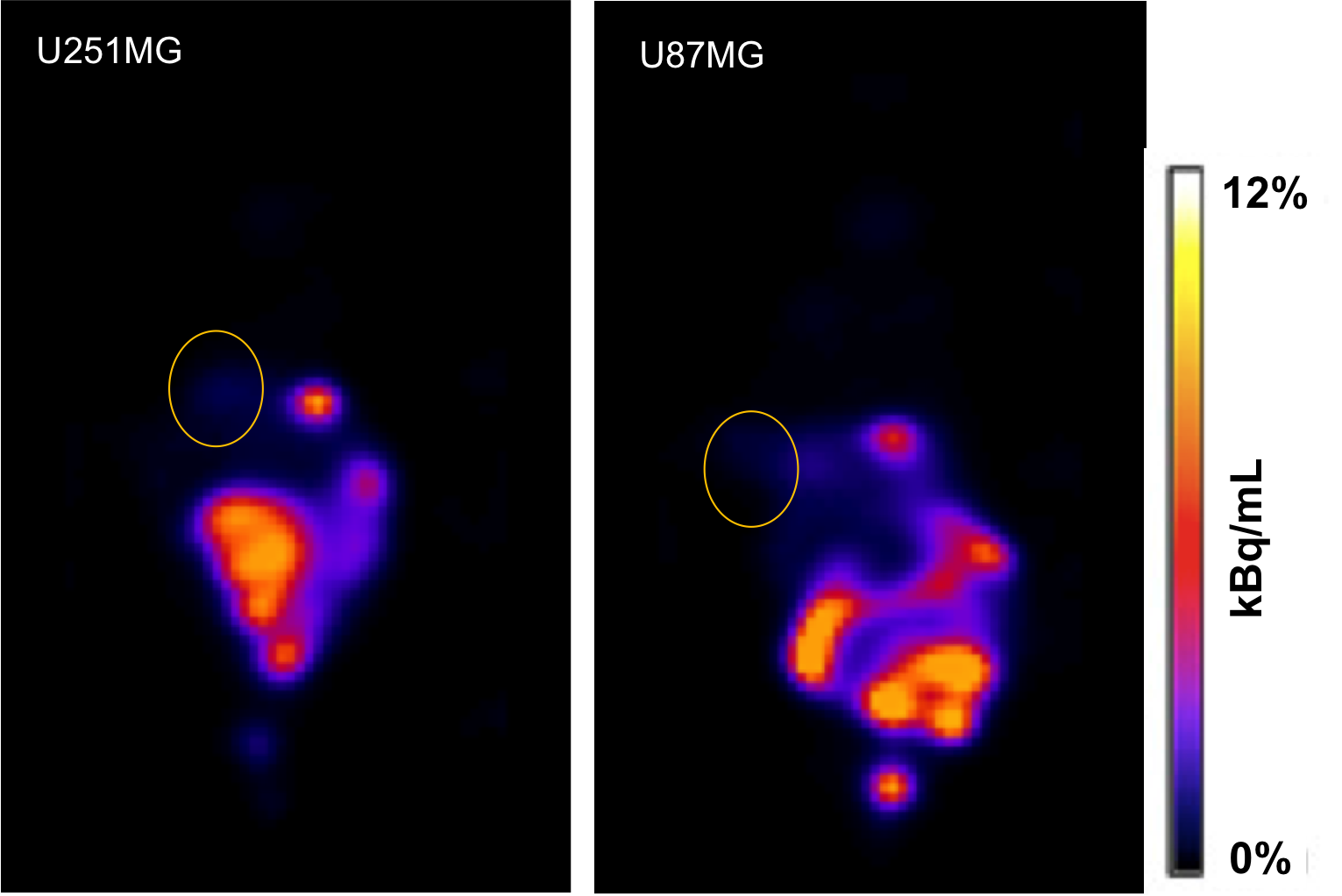


**Supplemental Fig. S2**: Schematic of experimental design of imaging experiments and representative MIP PET images of U251MG and U87MG xenograft-bearing mice 105 min after injection of [^18^F]olaparib.

**Supplemental Fig. S3**: A lack of correlation between injected radioactivity (0.28-13.89 MBq) and tumour uptake of [^18^F]olaparib in the U87MG and U251MG xenograft tumours.


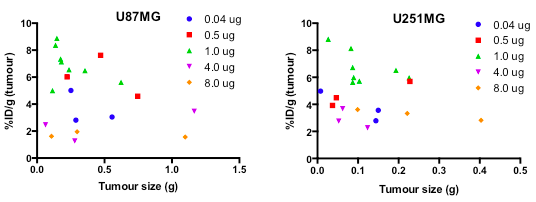


**Supplemental Fig. S4**: A lack of effect of tumour size (0.03-1.26 g) on tumour uptake of [^18^F]olaparib in the U87MG and U251MG xenograft tumours.

**Supplemental Fig. S5**: The effect of injected mass (μg, [^18^F]olaparib + [^19^F]olaparib) on overall olaparib accumulation (pmol/g) in the U87MG (*P*>0.05) and U251MG (*P*<0.01) xenograft tumours.


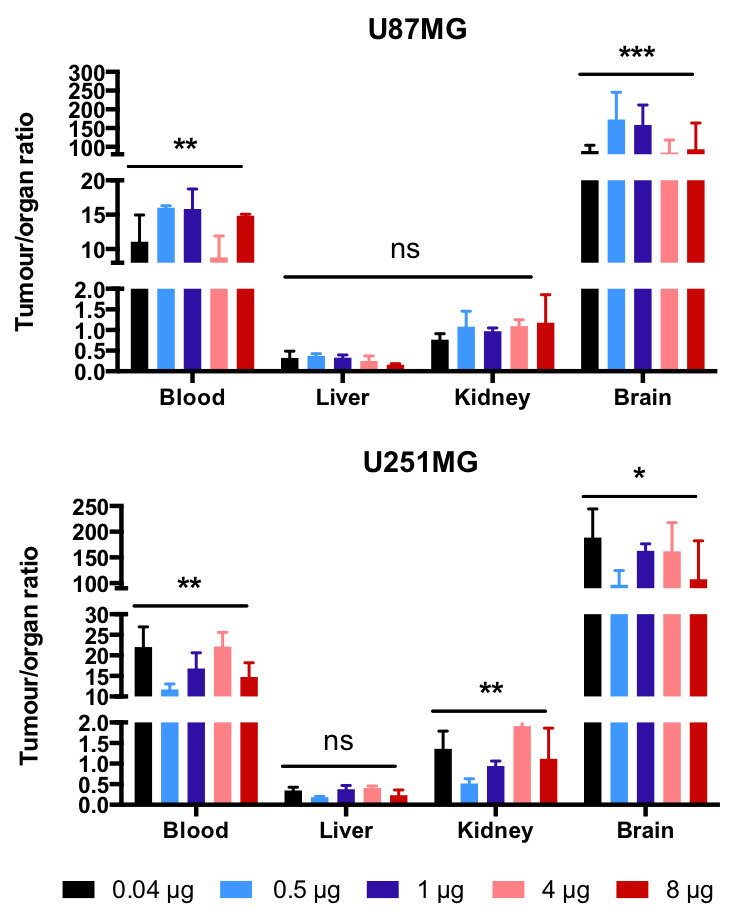


**Supplemental Fig. S6**: Tumour-to-organ ratio ([^18^F]olaparib, %ID/g) in the U251MG an U87MG xenograft bearing-mice. *^ns^P*>0.05; **P*<0.05; ***P*<0.01; ****P*<0.001.


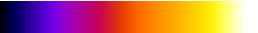


**Max**

**Min**


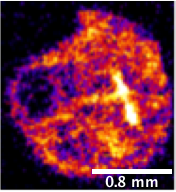


**U251MG**

Supplemental Fig S7: Autoradiography of U87MG tumour sections showing ^18^F localisation.
